# Evaluation of exoenzyme profiles of *Candida albicans* species isolated from females with vaginal candidiasis

**DOI:** 10.22034/cmm.2024.345112.1451

**Published:** 2023-12

**Authors:** Hasti Nouraei, Leila Razeghian Jahromi, Mehdi Ghaderian Jahromi, Kamiar Zomorodian, Keyvan Pakshir

**Affiliations:** 1 Department of Parasitology and Mycology, School of Medicine, Shiraz University of Medical Sciences, Shiraz, Iran; 2 Research Center for Psychiatry and Behavior Science, Department of Psychiatry, School of Medicine, Shiraz University of Medical Sciences, Shiraz, Iran; 3 Department of Radiology, School of Medicine, Medical Imaging Research Center, Shiraz University of Medical Sciences, Shiraz, Iran; 4 Basic Sciences in Infectious Diseases Research Center, School of Medicine, Shiraz University of Medical Sciences, Shiraz, Iran

**Keywords:** *Candida albicans*, Exoenzyme profiles, Hemolysin activity, Vaginitis

## Abstract

**Background and Purpose:**

The three most common causes of vaginitis are bacteria, yeast, and Protozoa. *Candida albicans* is one of the most common causes of vaginitis and commonly affects millions of females with different signs and symptoms. Secretion of exoenzymes from *Candida* species plays an important role in virulence and pathogenesis. Increasing our knowledge about the pathogenesis of candidiasis could help to design new anti-*Candida* drugs. This study aimed to evaluate the phospholipase, esterase, and hemolysin activities of the vaginal *Candida* isolates and their correlation with the presence of vulvovaginal candidiasis.

**Materials and Methods:**

In total, 119 *Candida albicans* isolates from vaginal candidiasis were enrolled in the study. Egg yolk agar, Tween 80 opacity medium, and blood agar plate assays were used for the determination of phospholipase, esterase, and hemolytic activities, respectively.

**Results:**

Based on the findings, 110 (92.44%) isolates showed phospholipase activity, 93 (78.2%) isolates were esterase producers, and 90 (75.6%) species had hemolytic activity.

**Conclusion:**

This study showed that most of the tested isolates had different enzymatic patterns. Discrimination of variations in the production of these exoenzymes among different *Candida* isolates may depend on *Candida* spp. pathogenicity and could be responsible for the severity of symptoms among the patients.

## Introduction

Female genital tract infections have an important effect on public health and have a high incidence rate among different age groups of females. Among these infections, vaginitis refers to inflammation of the vulva and/or vagina and is caused by different groups of pathogens, such as parasites (*Trichomonas*), bacteria (*Lactobacillus*), and fungi (*Candida*), and has different diagnostic criteria [ 1 ]. Trichomoniasis is a common parasitic sexually-transmitted infection that may have no symptoms in 64-90% of infected people, but symptomatic females have a greatly increased volume of vaginal discharge that may be green or yellow in color, frothy, and malodorous. Bacterial vaginosis is more common in black females, smokers, intravaginal product users, and sexually active females [ 2 ].

Vulvovaginal candidiasis (VVC) is a common clinical condition with symptoms and signs, such as itching, curd-like vaginal discharge, and erythema. It is the result of overgrowth and penetration of *Candida* species in the vaginal mucosa and induction of an inflammatory response [ 3 ]. *Candida* species are the part of normal flora of the vagina and about 70-75% of women experience VVC at least once in their lifetime while approximately 50% of females experience it multiple times [ 4 ]. In general, *Candida* species is now ranked as the primary cause of vaginal infections. Although different species of *Candida*, such as *C. glabrata*, *C. parapsilosis*, and *C. tropicalis* cause VVC, most often it is caused by *C. albicans*. Over 85-90% of the vaginal *Candida* isolates obtained from patients with VVC are *C. albicans* [ 5 ]. 

Many risk factors are relative to the increasing rate of VVC in females, such as hormonal fluctuation, like increased estrogen levels that occur during pregnancy and menstrual cycles, diabetes, immunologic alterations, sexual activity, increased vaginal glycogen production mechanism, sustained antibiotic use, and oral contraceptive consumption [ 6 ]. *Candida albicans* has a lot of virulence factors that contribute to pathogenesis, including the production of adhesion to host cells, biofilm formation, phenotypic switching, and the secretion of various exoenzymes, such as phospholipases, esterase, proteases, and hemolysin [ 7 ]. Phospholipases digest the host cell membranes for better attachment, invasion, infection development, and cell destruction [ 8 ].

The ability to obtain iron in mammalian hosts by pathogenic organisms plays an important role in causing infection by them, and since in humans, most of the iron is stored inside cells as ferritin or compounds with heme, the pathogens must use hemolysin-produce mechanism to obtain iron, especially during menstruation period; hence, this enzyme is very important for organism [ 8 ].

Esterase is another hydrolyzing enzyme that breaks down ester during a chemical reaction to acid and alcohol and it is important in the pathogenicity of *Candida* spp. [ 9 ]. The most cases of *Candida vaginitis* are caused by *C. albicans* species. Also, the presence of high enzymatic activity may be involved in severity of invasion and destroying host cells. All these could cause exacerbating different clinical sign and symptoms. This study aimed to provide a comparative analysis of phospholipase, esterase, and hemolysin activity among the *Candida albicans* vaginitis isolates in patients suffering from *Candida* vaginitis.

## Materials and Methods

### Yeast isolates

In this study, in total, 119 stocks of *C. albicans* species that were previously recovered from *Candida* vaginitis patients were examined [ 10 , 11 ]. They were identified to the species level by molecular method (polymerase chain reaction-restriction fragment length polymorphism) previously and kept at -20 °C. These isolates were sub-cultured on Sabouraud dextrose agar (Merck, Germany) before usage. 

### Phospholipase activity

The plate method was used for the evaluation of phospholipase activity according to Price et al. [ 12 ]. The medium was prepared by the addition of 58.4 g of NaCl and 5.5 g of CaCl_2_ to Sabouraud dextrose agar (Merck, Germany). The sterile egg yolk was centrifuged at 5,000 ×g for 30 min and 20 mL of supernatant was added to the cooled above-mentioned medium. The inocula was prepared (2 McFarland turbidity) and 10 μL yeast suspension was spot-inoculated on the plate medium and incubated at 37 °C for up to 5 days. A precipitate zone around the colonies was measured, and expressed as Pz value from 1 to 4 as follows: Pz 1 (negative), Pz 1+ (0.9–1), Pz 2+ (0.80–0.89), Pz 3+ (0.70–0.79), and Pz 4+ (≥0.69). The standard strain of *C. albicans* (ATCC10261) was used as a control.

### Esterase activity

Esterase activity was measured using the Tween 80 opacity test medium [ 13 ]. Moreover, 10 g of bactopepton, 5 g of NaCl, 0.1 g of Cacl2, 15g of agar, and 1,000 mL of distilled water were used for the preparation of the medium. After autoclaving, 5 ml of autoclaved Tween 80 was added to the medium and distributed in sterile 10 cm plates. Subsequently, 10 μL of *Candida* suspension (10^6^ cells/ml) was inoculated as a spot on the plates and kept at 35 ° C for 10 days. The colony diameter (a) and the diameter of the colony plus precipitation zone (b) were measured to calculate the zone of esterase activity.

### Hemolysin activity

Blood plate assay was used as explained by Yigit and Aktas for hemolytic activity [ 14 ]. By the addition of 7% v/v of fresh sheep blood and 3% w/v of glucose to Sabouraud dextrose agar, the media was prepared. Afterward, 2 McFarland turbidity of yeast samples suspension was prepared and 10 μL was spotted on medium and incubated at 37 °C for 48 h. For hemolytic activity, a ring of lysis around the colonies was considered complete: in case of a totally translucent ring; beta, incomplete: in case of a greenish-black halo; alpha, and no hemolysis: gamma or none. *Candida albicans* ATCC10261 was used as a control.

### Statistical Analysis

Results were analyzed in the SPSS software (version 22). 

### Ethical approval

This project was in accordance with the ethical principles and the national norms and standards for the conduction of Medical Research in Iran and has been approved by the Research Ethics Committee of Shiraz University of Medical Sciences, Shiraz, Iran (IR. SUMS.REC.1390.3755).

## Results and Discussion

Vulvovaginal candidiasis corresponds to an infection that is the second cause of vaginitis and leads females to frequent medical visits in different parts of the world. Due to the development of gynecological examinations, many females present with VVC which is associated with horrible and painful signs and symptoms for most patients. *Candida* species are a normal commensal in the human body, including the female genital tract. Abnormal growth of yeasts in the region of the female genital tract causes VVC [ 15 ]. Its growth is controlled and limited by the immune system and the dynamics of complex bacterial microbiome. 

*Candida albicans* is responsible for the majority of symptomatic VVC. Many factors have been attributed to the pathogenic potentials of *C. albicans*, such as the secretion of various enzymes, phenotypic switching, biofilm formation, and yeast-to-hypha transition. According to previous studies, *C. albicans* isolates could secrete higher amounts of these enzymes, compared to non-*albicans Candida*, and the ability of exo-enzyme production was an important virulence factor in *C. albicans* isolates [ 8 , 16 ]. Ability of this organism to produce extracellular enzymes may play a role in its pathogenicity, invasion, and destruction of host tissues and also could create and develop clinical signs. 

In addition, there is a significant statistical correlation between enzymatic production and resistance of yeasts to azole antifungal agents [ 18 ]. *Candida albicans* use their phospholipase and hemolysin enzyme activity to invade to host tissue. Furthermore, the esterase enzyme, as a hydrolyzing enzyme, plays an important role in *Candida* invasion by breaking down fats. Although *Candida* has the ability to produce these enzymes, their amount and strength vary among the different species, which may be due to the source of their isolation [ 18 , 19 ]. Phospholipase secretions also help the organism to escape from host defense mechanisms [ 8 ]. 

**Figure 1:**
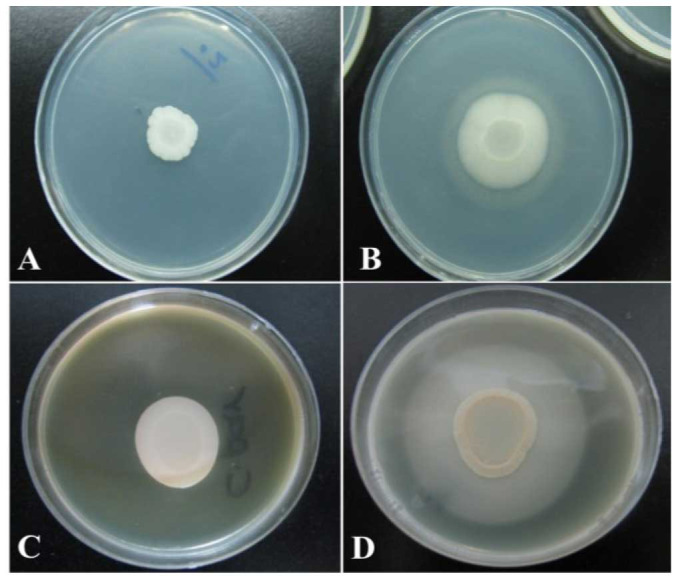
Esterase and Phospholipase production of *Candida albicans* isolates. A) No esterase production was observed. B) Positive activity of esterase was observed. C) No phospholipase production was observed. D) Dense white zone of precipitation around the colony indicated phospholipase activity.

In this study, analysis of Egg yolk agar test data showed that most of the samples had phospholipase activity in different ranges (Figure 1). In total, 110 (92.44%) out of 119 samples had phospholipase activity, 98 species (82.3%) showed +4 positive for phospholipase activity, and only 9 isolates (7.56%) had no phospholipase activity. More details are summarized in Table 1. 

**Table 1:** Frequency of phospholipase activity and esterase activity in different isolates of *Candida albicans*

		Exoenzyme Activity				
		Negative	1+	2+	3+	4+
**Esterase activity**	Number (%)	26 (21.8)	0(0)	1(0.84)	4(3.3)	88(73.9)
**Phospholipase activity**	Number (%)	9 (7.56)	0(0)	4(3.36)	8(6.7)	98(82.3)

In a study performed by Sachin et al. [ 20 ], 92.3% of *C. albicans* isolates demonstrated phospholipase activity, but in another study conducted by Shirkhani et al. [ 21 ], only 29.4% of *C. albicans* isolates had phospholipase activity. Therefore, the amount of this secretion could be important in tissue invasion for organisms and could aggravate clinical symptoms in infected people. 

Hemolytic enzymes are another important factor in the pathogenicity of *Candida* that enable the pathogen to extract iron from hemoglobin or hemin molecules in host cells [ 22 ]. Due to the vaginal environment covers with large amounts of blood during menstruation, this condition can further activate this enzyme in *Candida* species and provide iron from the blood. In the present study, 75.6%, 67.2% (80), 8.4% (10), and 24.4% (29) of isolates had hemolytic activity, beta-hemolytic activity, alpha-hemolytic activity, and gamma hemolytic activity with no hemolytic activity, respectively. 

In a study carried out by Sachin et al. [ 20 ], hemolytic activity was detected in 100% and 94.8% of samples of *C. albicans*, respectively; however, these data were not in line with those of the present study. Esterase activity is another virulence factor in *C. albicans* which contributes to host tissue invasion by disruption of host cell membranes [ 9 ]. In the present study, the evaluation of data from the Tween 80 opacity test demonstrated that 93 (78.2%) out of 119 studied samples had esterase activity (Figure 1), and 88 isolates (73.9%) showed +4 positive esterase activity. Moreover, 26 isolates (21.8%) had no esterase activity (Table 1). It should be noted that these virulence factors play a crucial role in the pathogenesis. Detection of virulence factors, like phospholipase, esterase, and hemolysin activity will help provide sufficient data for a better understanding of the relationship between infection and the isolated *Candida* species. Besides, the severity of extracellular enzymes may play a role in the severity of signs and symptoms of vulvovaginal candidiasis. Knowledge about these virulence factors can be used to improve disease management and treatment. However, further studies in a higher number of species are required to confirm these findings.

## Conclusion

Vulvovaginitis is considered a complex infection that occurs due to different microorganisms in the vagina. *Candida albicans* is one of the most common reasons for vaginitis. Virulence factors, like phospholipase, esterase, and hemolysin activity, play important roles in the pathogenicity of *C. albicans*. These findings should help the effective administration of *Candida* vaginitis. Continued observation would greatly support the management of this largely opportunistic infection. Further research aimed at the characterization of possible risk factors as well as preventive therapy and treatment is suggested.
